# The importance of staying within range: associations between tacrolimus intrapatient variability and kidney transplant outcomes

**DOI:** 10.3389/fimmu.2026.1739104

**Published:** 2026-01-29

**Authors:** Louise Benning, Lisa Wittig, Marvin Reineke, Maarten Busse, Claudius Speer, Martin Zeier, Christian Morath, Thuong Hien Tran, Bernd Döhler

**Affiliations:** 1 Department of Nephrology, University Hospital Heidelberg, Heidelberg, Germany; 2 Department of Cardiology, Angiology and Pneumology, University Hospital Heidelberg, Heidelberg, Germany; 3 Department of Nephrology and Hypertension, Klinikum Nuremberg, Paracelsus Medical University, Nuremberg, Germany; 4 Department of Transplantation Immunology, Institute of Immunology, University of Heidelberg, Heidelberg, Germany

**Keywords:** graft survival, intrapatient variability, kidney transplantation, rejection, tacrolimus

## Abstract

**Background:**

Kidney transplantation remains the treatment of choice for end-stage kidney disease. Maintaining immunosuppression within the appropriate therapeutic range is essential to prevent rejection and ensure long-term graft survival. This study evaluated the clinical relevance of different tacrolimus exposure metrics and their association with post-transplant outcomes in a real-world kidney transplant cohort.

**Methods:**

Of 881 adult deceased-donor kidney transplants performed between 2011 and 2020 at Heidelberg University Hospital, 372 recipients with a functioning graft at day 180 met the inclusion criteria and were included in the final analysis. Tacrolimus trough levels between days 90 and 180 were used to calculate different exposure metrics, including intrapatient variability (IPV), maximum quotient, minimum trough levels, and the area under the curve (AUC) to approximate time spent below the therapeutic threshold. Outcomes analyzed included 5-year death-censored graft survival, 5-year overall graft and patient survival, 3-year rejection-free survival, and 3-year donor-specific antibody (DSA)-free survival.

**Results:**

High tacrolimus IPV (≥30%) was associated with a 2.4-fold increased risk of rejection (95% CI 1.3–4.6; *P*=0.009). A high quotient (≥3.0) was linked to a 2.3-fold higher risk of rejection (95% CI 1.2–4.5; *P*=0.014) and showed a trend toward worse graft survival (HR 1.9; 95% CI 1.0–3.6; *P*=0.050). Prolonged time below target levels (<6 ng/mL; AUC ≥0.2) was significantly associated with increased risks of graft failure (HR 3.4; 95% CI 1.9–6.0; *P*<0.001), death-censored graft failure (HR 4.0; 95% CI 2.1–7.6; *P*<0.001), and patient death (HR 4.1; 95% CI 1.6–10.3; *P*=0.003). Minimum trough levels <5 ng/mL were also strongly associated with adverse outcomes, including graft failure (HR 3.2; 95% CI 1.8–5.7; *P*<0.001), death-censored graft failure (HR 4.0; 95% CI 2.1–7.8; *P*<0.001), patient death (HR 3.4; 95% CI 1.3–9.0; *P*=0.012), and rejection (HR 2.5; 95% CI 1.3–4.6; *P*=0.005). No association was observed between any of the exposure metrics and the development of DSAs.

**Conclusions:**

Multiple markers of tacrolimus underexposure were independently associated with poor post-transplant outcomes. These findings underscore the critical importance of maintaining tacrolimus levels within the target therapeutic range during the early post-transplant period to optimize long-term kidney transplant outcomes.

## 1 Introduction

Kidney transplantation provides patients with end-stage kidney disease the most effective option for achieving long-term survival and improved quality of life, while also being the most cost-efficient form of renal replacement therapy (1). Compared to dialysis, kidney transplantation has consistently been associated with significantly lower mortality and reduced cardiovascular risk, and these benefits even increase over time (2). Total adult kidney transplant reached a high of 27, 351 procedures performed in the United States according to Organ Procurement and Transplantation Network (OPTN) and Scientific Registry of Transplant Recipients (SRTR) 2023 data (1). Following kidney transplantation, lifelong immunosuppressive maintenance therapy is essential to prevent rejection and maintain allograft function (3, 4). Current immunosuppressive protocols have resulted in very low acute rejection rates (5), ranging from 5.1% in recipients aged 65 and older to 8.0% in those aged 18–34 during the first post-transplant year, according to 2023 OPTN/SRTR data (1). In the early post-transplant period, most patients receive a tacrolimus-based regimen in combination with mycophenolate, either with corticosteroids (68.8%) or without them (24.7%) (1).

Tacrolimus has emerged as the cornerstone of maintenance immunosuppression in kidney transplantation, owing to its superior efficacy in preventing acute rejection compared to cyclosporine, which itself had revolutionized the field following its approval by the Food and Drug Administration (FDA) in 1983 (6). First identified as FK506 in 1984 and introduced clinically in 1989, tacrolimus quickly gained recognition for its potent immunosuppressive effects (7, 8). Following initial use in high immunological risk and multiorgan recipients (9), robust randomized controlled trials in kidney transplantation demonstrated improved graft outcomes and established tacrolimus as the preferred calcineurin inhibitor (10–12). Given its narrow therapeutic window, complex pharmacokinetics, and high inter- and intrapatient variability, tacrolimus requires close therapeutic drug monitoring (TDM) (13–15). Tacrolimus exposure is usually monitored using target trough concentrations (C_0_) in whole blood, which correlate well with the area under the concentration-time curve (AUC_0-12_) (6). While C_0_ levels are routinely used to guide dosing and maintain drug levels within established therapeutic ranges, achieving consistent and adequate exposure remains a major challenge in clinical practice (16, 17). Fluctuations in tacrolimus levels and subsequent low systemic exposure have been linked to an increased risk of immune-mediated complications, including acute rejection and graft loss (18, 19).

Among the various factors influencing tacrolimus pharmacodynamics, intrapatient variability (IPV), typically quantified by the coefficient of variation (CV) of C_0_ levels during stable dosing periods, has emerged as a critical predictor of transplant outcomes (20–22). High tacrolimus IPV has been independently associated with an increased risk of late acute rejection, donor-specific antibody (DSA) formation, chronic allograft dysfunction, and graft loss (22). IPV can be affected by multiple factors, including drug-drug interactions, food intake, gastrointestinal disturbances, medication formulation, generic substitution, assay variability, and genetic polymorphisms such as those in CYP3A5, which influence tacrolimus metabolism and clearance (20, 23). Importantly, high IPV has also been associated with behavioral factors, with medication nonadherence recognized as a predominant and modifiable contributor (20).

Most studies evaluating the impact of tacrolimus IPV on graft outcomes have focused on tacrolimus levels during 6–12 months post-transplant or even later to calculate for IPV (22, 24–29), most likely to avoid early confounders such as early rejection episodes and fluctuating drug absorption during hospitalizations (20). However, this strategy may overlook a critical window in which actionable variation in tacrolimus exposure could already influence long-term outcomes. In this study, we therefore aimed to evaluate the clinical relevance of tacrolimus exposure metrics during the earlier 3–6 month period. Beyond IPV, we examined additional parameters including maximum quotient, minimum levels, and area under the curve (AUC) to approximate time spent below the therapeutic threshold in a real-world cohort of kidney transplant recipients. By analyzing their associations with key post-transplant outcomes including rejection, graft loss, and patient survival, we sought to identify early, actionable markers to guide individualized immunosuppressive management.

## 2 Materials and methods

### 2.1 Study design

Patients were eligible for inclusion if they met all of the following criteria: (i) age ≥18 years at the time of transplantation; (ii) kidney-only transplantation performed at Heidelberg University Hospital between 2011 and 2020; (iii) functioning kidney allograft at day 180 post-transplantation; (iv) maintenance immunosuppression with a standardized triple regimen consisting of twice-daily tacrolimus, mycophenolic acid, and corticosteroids; (v) availability of tacrolimus trough level measurements between days 90 and 180 post-transplantation. Patients were excluded if they met any of the following criteria: (i) change in immunosuppressive regimen during the 90-180-day post-transplant observation period; (ii) insufficient documentation of tacrolimus trough levels during the observation period; (iii) failure to meet one or more of the inclusion criteria.

Multiple outcomes were assessed, including 5-year death-censored graft survival, 5-year overall graft survival, 5-year patient survival, 3-year biopsy-proven rejection, and 3-year *de novo* donor-specific antibody (dnDSA) formation. The study was approved by the ethics committee of the University of Heidelberg (S-119/2024), registered in the German Clinical Trials Register (DRKS00033736), and conducted in accordance with the Declaration of Helsinki.

### 2.2 Tacrolimus exposure metrics

Tacrolimus has been the preferred calcineurin inhibitor at our center since late 2018, with target trough levels of 8–10 ng/mL (weeks 1-6), 5–8 ng/mL (until month 6), and 4–6 ng/mL thereafter, tailored to the recipient’s immunological risk at transplantation, as described previously (30). Tacrolimus levels were monitored using a validated liquid chromatography-tandem mass spectrometry (LC-MS/MS) assay (31).

Tacrolimus intrapatient variability (IPV) was assessed using all collected trough levels (C_0_) between days 90 and 180 post-transplant, a period representing an early but clinically stable maintenance phase. Accordingly, IPV was interpreted as a measure of real-world tacrolimus exposure variability during maintenance therapy rather than as variability under strictly unchanged dosing. The time window was selected to minimize perioperative confounders while preceding the post-transplant periods most commonly evaluated in prior studies (>6 months). IPV was calculated as the standard, unweighted coefficient of variation (CV%), defined as the standard deviation divided by the arithmetic mean of C_0_ levels, multiplied by 100. A time-weighted CV was not used. Based on prior studies, outcomes were analyzed using a threshold of ≥30% to define a high IPV (24, 32).

To further characterize tacrolimus variability, we applied a simple and clinically practical approach by calculating the maximum quotient between the highest and lowest tacrolimus trough levels, as described previously (24), now during the 90- to 180-day post-transplant period. In addition, we examined the associations of both mean and minimum tacrolimus trough levels with outcomes during the observation period.

To quantify the duration and extent of tacrolimus underexposure, we calculated the area under the curve (AUC) for trough levels below the therapeutic threshold of 6 ng/mL using the trapezoidal rule, normalized by the time between the first and last measurement. This metric provides a time-weighted estimate of prolonged subtherapeutic exposure, which recent evidence suggests is clinically meaningful in assessing the adequacy of immunosuppression in calcineurin inhibitor-based regimens (33, 34).

### 2.3 Statistical analysis

The impact of tacrolimus IPV, maximum quotient, minimum trough levels, and AUC on long-term transplant outcomes was assessed using Kaplan-Meier survival analysis. Mean survival times and standard errors reported for time-to-event outcomes represent estimates derived from Kaplan-Meier analysis and do not assume normal distribution of the underlying data. Group differences were evaluated using the Mantel-Cox log-rank test for trend.

To adjust for potential confounders, multivariable Cox regression analysis was performed, including the following dichotomous variables: transplant year (<2015), transplant number (retransplant), modality of transplant (living donor), recipient and donor sex (female), recipient and donor age (≥60 years), cause of donor death (cardiovascular), donor history of hypertension (yes), preemptive transplantation (yes), time on dialysis (≥5 years), cold ischemia time (>18 hours for deceased donors), HLA A+B+DR mismatches (>4), underlying kidney disease (non-glomerular), use of induction therapy (yes), presence of DSA at time of transplant (yes), prior desensitization (yes), panel reactive antibody level (>0%), use of antihypertensive medication (yes), and treatment for diabetes (yes). Variables with *P* values >0.2 were excluded using a backward stepwise elimination, and interactions between variables were assessed. Results are presented as hazard ratios (HRs) and 95% confidence intervals (CIs).

Missing data were handled using a complete-case approach without imputation. Patients with insufficient tacrolimus trough level measurements during the predefined observation period were excluded according to the inclusion criteria. Extreme tacrolimus trough values were reviewed by chart review and excluded if deemed implausible or attributable to measurement or documentation errors. Given the correlated nature of the tacrolimus exposure metrics and clinical outcomes, formal adjustment for multiple comparisons was not applied. The analyses were conducted in an exploratory, hypothesis-generating framework, with consistency of associations across related endpoints emphasized rather than strict control for multiplicity.

All statistical analyses were conducted using IBM SPSS Statistics for Windows, version 29.0 (IBM Corp., Armonk, NY, USA), with significance set at *P*<0.05.

## 3 Results

### 3.1 Study cohort

Between 2011 and 2020, a total of 881 adult deceased donor kidney-only transplants were performed at Heidelberg University Hospital. As tacrolimus became the standard calcineurin inhibitor at our center in 2018, previously being reserved for high immunological risk transplants only, 372 patients met the inclusion criteria for further analysis (**Supplementary Figure S1**).

Of the included patients, 65.1% received deceased-donor kidney transplants, and 75.8% of the overall cohort were undergoing their first kidney transplant. Recipients were predominantly male (55.9%) and under 60 years of age (76.9%), while most organ donors were also younger than 60 years (67.5%). Most organs were procured from donors with cerebrovascular cause of death (62.0%). Prior to transplantation, 47.6% of recipients had a dialysis vintage exceeding five years, whereas 52.4% had been on dialysis for less than five years or underwent preemptive transplantation. **Table 1** displays 5-year death-censored graft survival rates stratified by baseline characteristics. Five-year death-censored graft survival differed significantly by modality of transplant (*P*=0.001), number of transplant (*P*=0.040), donor age (*P*=0.008), cause of donor death (*P*=0.015), and dialysis vintage (*P*=0.039), whereas other baseline characteristics showed no significant association (**Table 1**). These variables, along with other clinically relevant covariates, were included in multivariable Cox regression models to adjust for potential confounding.

**Table 1:** Kaplan-Meier estimates of five-year death-censored graft survival by baseline clinical and immunological characteristics.

Variable	Category (Percentage)	5-Year death-censored graft survival ± SE	Log-rank *P*-value
**Modality of Transplant**	**Deceased Donor (65.1%)** **Living Donor (34.9%)**	**0.839 ± 0.025** **0.957 ± 0.019**	0.001(**)
**Number of Transplant**	**First Transplant (75.8%)** **Re-Transplant (24.2%)**	**0.904 ± 0.019** **0.813 ± 0.044**	0.040(*)
Recipient Sex	Male (55.9%) Female (44.1%)	0.880 ± 0.024 0.883 ± 0.026	0.93
Recipient Age	<60 years of age (76.9%) ≥60 years of age (23.1%)	0.898 ± 0.019 0.824 ± 0.045	0.092
**Donor Age**	**<60 years of age (67.5%)** **≥60 years of age (32.5%)**	**0.915 ± 0.018** **0.803 ± 0.041**	0.008(**)
**Cause of Donor Death** **(deceased donor tx)**	**Cerebrovascular (62.0%)** **Other (38.0%)**	**0.789 ± 0.036** **0.922 ± 0.028**	0.015^(*)^
Cold Ischemia Time (deceased donor tx)	≤18 hours (82.5%) >18 hours (17.5%)	0.848 ± 0.034 0.848 ± 0.071	0.97
**Dialysis Vintage**	**preemptive or ≤5 y (52.4%)** **>5 y (47.6%)**	**0.914 ± 0.022** **0.846 ± 0.029**	0.039(*)
HLA-mismatches (Deceased Donor tx)	0–4 (88.2%) 5–6 (11.2%)	0.851 ± 0.026 0.728 ± 0.103	0.12
Pretransplant AB (CDC or Luminex)	Negative (16.4%) Positive (83.6%)	0.856 ± 0.052 0.885 ± 0.019	0.83
Donor-specific Antibodies	No (81.0%) Yes (19.0%)	0.886 ± 0.020 0.856 ± 0.045	0.57
Desensitization prior to Transplantation	No (51.9%) Yes (48.1%)	0.850 ± 0.032 0.856 ± 0.033	0.84

Values represent Kaplan-Meier estimates of 5-year death-censored graft survival ± standard error stratified by key transplant-related variables. Survival differences between groups were assessed using the log-rank test. Statistically significant associations (*P*<0.05) are indicated in bold.

AB, antibodies; CDC, complement-dependent cytotoxicity; d, days; HLA, human leukocyte antigen; SAB, single antigen bead; Tx, transplant; **P<0.01; *P<0.05.

A total of 2,505 tacrolimus trough levels were recorded for the entire cohort. Among these, 255 patients (68.5%) had at least three tacrolimus trough levels documented within the specified time frame (**Supplementary Figure S2A**). Notably, patients with more frequent trough level measurements (>8 measurements) had significantly lower 5-year death-censored graft survival (*P*<0.001, **Supplementary Figure S2B**), likely reflecting closer monitoring in the context of early post-transplant complications.

### 3.2 High tacrolimus intrapatient variability is associated with an increased risk for rejection

**Figure 1:**
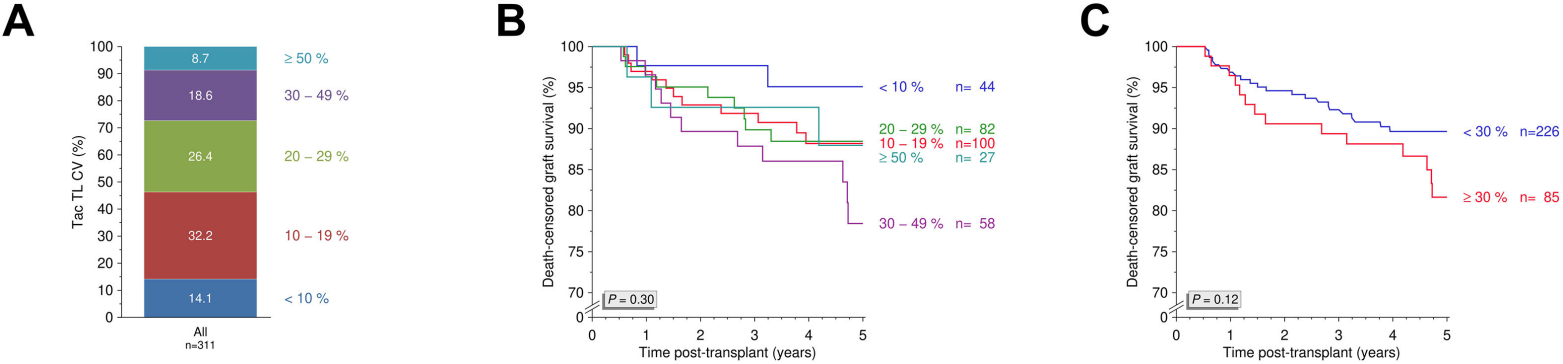
Tacrolimus intrapatient variability and five-year death-censored graft survival. **(A)** Distribution of tacrolimus intrapatient variability (IPV) in the study cohort. **(B)** Five-year death-censored graft survival across different tacrolimus IPV thresholds. **(C)** Five-year death-censored graft survival between patients with high IPV ≥30% (N=85) and low IPV <30% (N=226). N, number; Tac, tacrolimus; TL, trough level.

**Figure 2:**
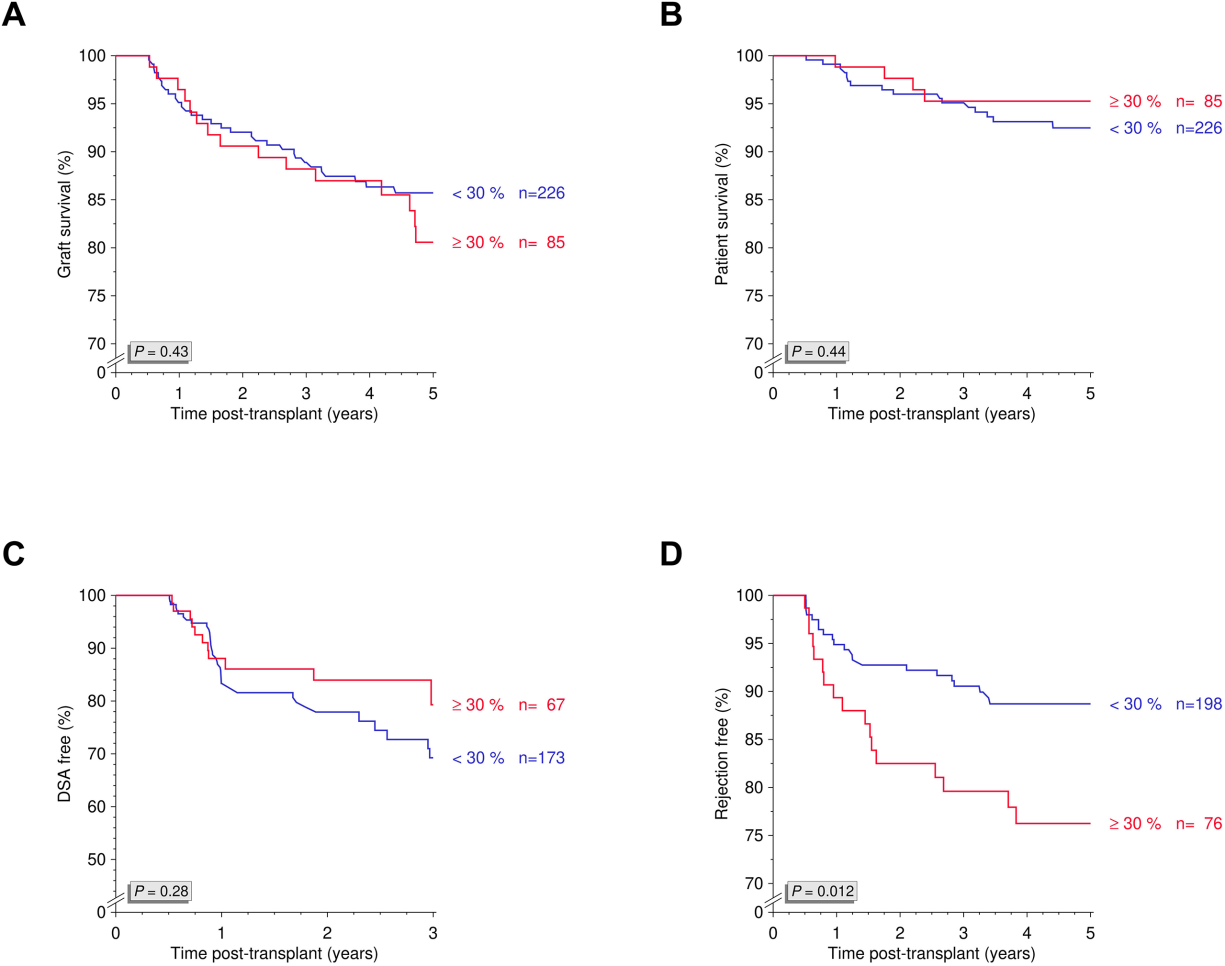
Tacrolimus intrapatient variability and overall graft survival, patient survival, DSA-free survival and rejection-free survival. Kaplan-Meier analyses of **(A)** overall graft survival, **(B)** patient survival, **(C)** DSA-free survival, and **(D)** rejection-free survival in patients with high (≥30%) and low (<30%) tacrolimus intrapatient variability. DSA, donor-specific antibodies; N, number.

Tacrolimus IPV varied widely across the cohort, with 27.3% of patients exhibiting an IPV ≥30% (**Figure 1A**). We further assessed 5-year death-censored graft survival using various other IPV thresholds and found no statistically significant differences (*P*=0.30; **Figure 1B**). When applying the ≥30% threshold to define high IPV, no significant difference in 5-year death-censored graft survival was observed between groups (*P*=0.12; **Figure 1C**). Similarly, Kaplan-Meier analyses showed no significant differences between the two groups in overall graft survival, patient survival, or DSA-free survival (all *P*>0.10; **Figures 2A–C**). However, rejection-free survival was significantly better among patients with IPV <30% (*P*=0.012; **Figure 2D**).

In multivariable Cox regression analysis, high tacrolimus intrapatient variability (IPV ≥30%) was independently associated with an increased risk of biopsy-proven rejection within three years post-transplant (HR 2.40, 95% CI 1.25–4.60; *P*=0.009). In contrast, no independent associations were observed with overall graft failure (HR 1.07, 95% CI 0.56–2.04; *P*=0.84), death-censored graft failure (HR 1.33, 95% CI 0.67–2.65; *P*=0.42), patient death (HR 0.57, 95% CI 0.18–1.80; *P*=0.34), or dnDSA formation (HR 1.04, 95% CI 0.51–2.12; *P*=0.92; **Supplementary Table S1**).

### 3.3 Large tacrolimus trough level fluctuations indicate increased risk of rejection and graft failure

**Figure 3:**
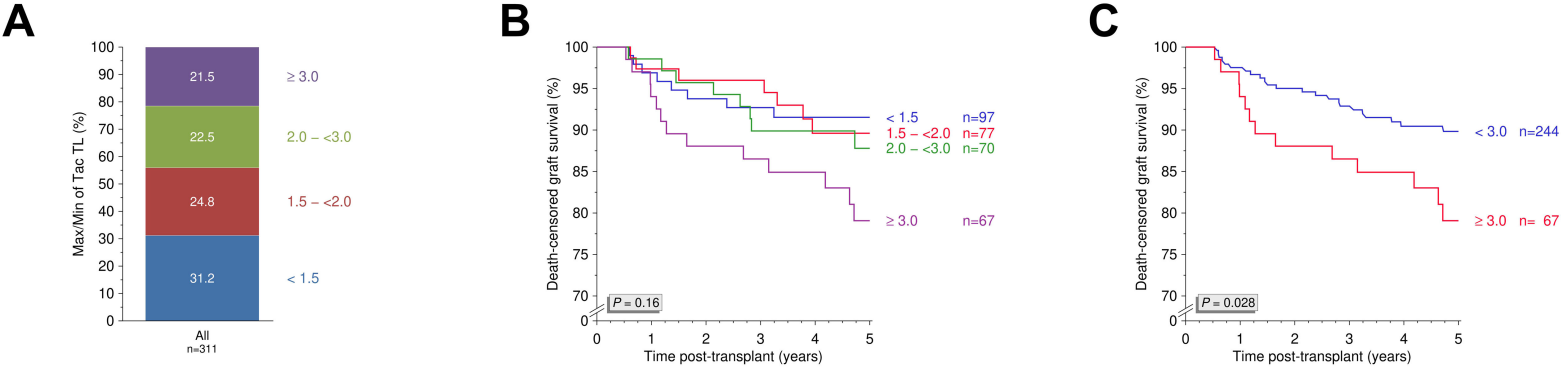
Maximum/minimum quotient of tacrolimus trough levels and five-year death-censored graft survival. **(A)** Distribution of maximum/minimum quotients between highest and lowest tacrolimus trough levels recorded between days 90 and 180 post-transplant in the study cohort. **(B)** Five-year death-censored graft survival across different quotients with identification of a clinically relevant quotient ≥3.0. **(C)** Five-year death-censored graft survival in patients with a quotient ≥3.0 (N=67) and <3.0 (N=244). N, number; Tac, tacrolimus; TL, trough level.

**Figure 4:**
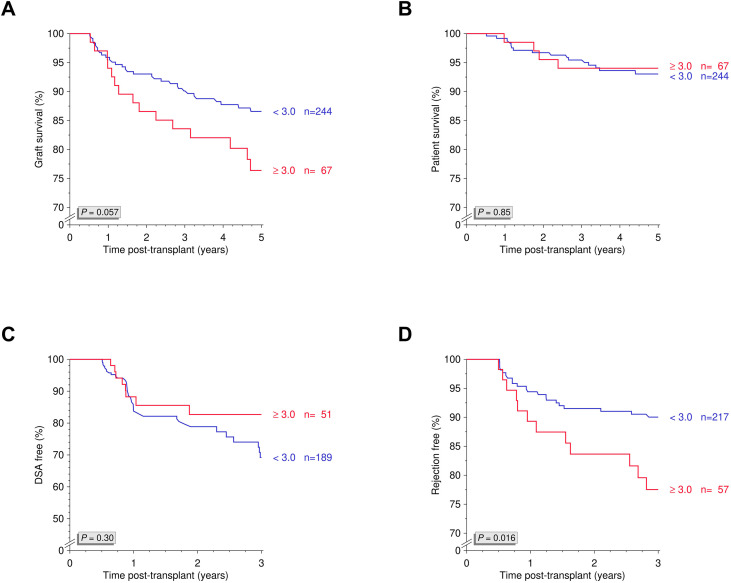
Maximum/minimum quotient of tacrolimus trough levels and overall graft survival, patient survival, DSA-free survival and rejection-free survival. Kaplan-Meier analyses of **(A)** overall graft survival, **(B)** patient survival, **(C)** DSA-free survival, and **(D)** rejection-free survival stratified by high versus low maximum/minimum quotient of tacrolimus trough levels between days 90 and 180 post-transplant. DSA, donor-specific antibodies; N, number.

To further evaluate tacrolimus variability, we calculated the maximum quotient between the highest and lowest trough levels during the observation period and stratified patients accordingly (**Figure 3A**). A cutoff ≥3.0 was identified as clinically relevant (**Figure 3B**) and was associated with significantly reduced 5-year death-censored graft survival (*P*=0.028; **Figure 3C**). In additional Kaplan-Meier analyses, no significant differences were observed between groups for overall graft survival, patient survival, or DSA-free survival (all *P*>0.05, **Figures 4A–C**). However, rejection-free survival was significantly worse in patients with a high quotient of maximum/minimum tacrolimus trough levels (*P*=0.016; **Figure 4D**).

Multivariable Cox regression analysis demonstrated that pronounced tacrolimus trough level fluctuations (maximum/minimum quotient ≥3.0) were independently associated with an increased risk of biopsy-proven rejection (HR 2.32, 95% CI 1.19–4.53; *P*=0.014) and showed a trend towards overall graft failure (HR 1.90, 95% CI 1.00–3.61; *P*=0.050). No significant associations were observed with death-censored graft failure (HR 1.90, 95% CI 0.95–3.81; *P*=0.069), patient death (HR 1.19, 95% CI 0.32–4.35; *P*=0.79), or dnDSA development (HR 0.92, 95% CI 0.42–2.00; *P*=0.83; **Supplementary Table S2**).

### 3.4 Prolonged time below the tacrolimus target range is associated with poorer graft outcomes

**Figure 5:**
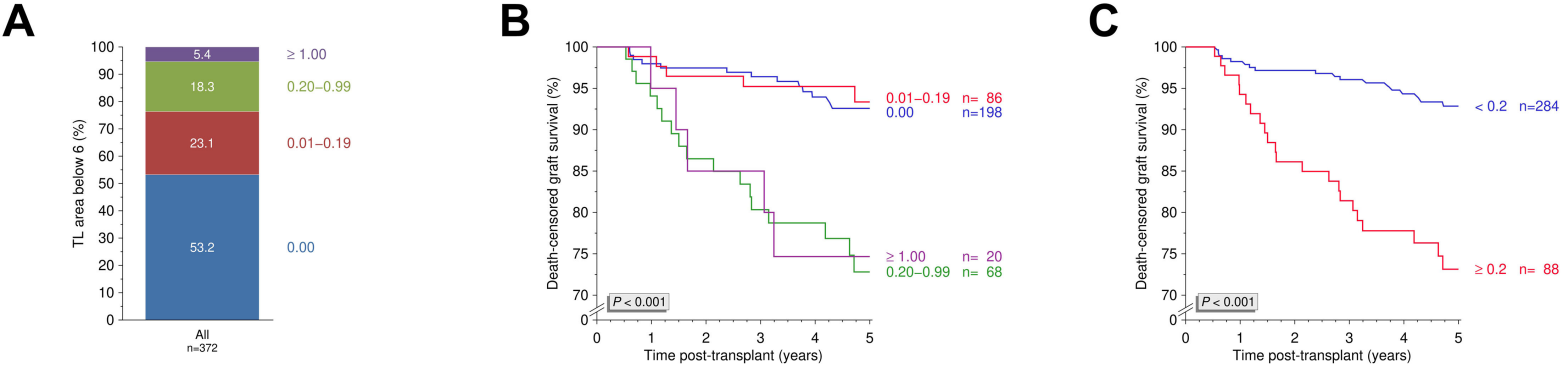
Normalized AUC for trough levels <6 ng/mL and five-year death-censored graft survival. **(A)** Distribution of normalized AUC values for tacrolimus trough levels <6 ng/mL between days 90 and 180 post-transplant in the study cohort. **(B)** Five-year death-censored graft survival across different normalized AUCs with identification of a clinically relevant AUC of 0.2. **(C)** Five-year death-censored graft survival in patients with an AUC ≥0.2 (N=88) and <0.2 (N=284). AUC, area under the curve; N, number; TL, trough level.

**Figure 6:**
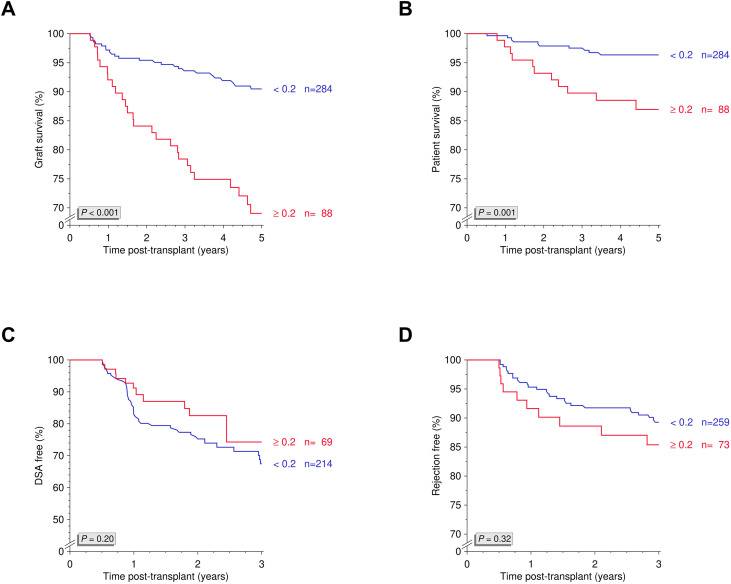
Normalized AUC for trough levels <6 ng/mL and overall graft survival, patient survival, DSA-free survival and rejection-free survival. Kaplan-Meier analyses of **(A)** overall graft survival, **(B)** patient survival, **(C)** DSA-free survival, and **(D)** rejection-free survival in patients stratified by normalized AUC for tacrolimus trough levels <6 ng/mL. AUC, area under the curve; DSA, donor-specific antibodies; N, number.

To assess prolonged underexposure to tacrolimus, we calculated a normalized AUC for trough levels <6 ng/mL between days 90 and 180 post-transplant. An AUC ≥0.2, indicating an extended time spent below therapeutic threshold, was observed in 23.7% of the cohort (**Figure 5A**) and was considered a clinically relevant cutoff for subsequent analyses (**Figure 5B**). Patients with an AUC ≥0.2 showed significantly poorer 5-year death-censored graft survival compared with those with an AUC <0.2 (*P*<0.001; **Figure 5C**). Additionally, overall graft survival (*P*<0.001; **Figure 6A**) and patient survival (*P*=0.001; **Figure 6B**) differed significantly between groups. No significant associations were observed for rejection-free or DSA-free survival (both *P*>0.10; **Figures 6C, D**).

In multivariable Cox regression analysis, prolonged time below the tacrolimus target range (AUC ≥0.2) was strongly and independently associated with adverse long-term outcomes, including overall graft failure (HR 3.40, 95% CI 1.91–6.03; *P*<0.001), death-censored graft failure (HR 4.03, 95% CI 2.13–7.60; *P*<0.001), and patient death (HR 4.05, 95% CI 1.60–10.3; *P*=0.003). No independent associations were observed with biopsy-proven rejection (HR 1.47, 95% CI 0.75–2.87; *P*=0.26) or dnDSA formation (HR 0.74, 95% CI 0.38–1.44; *P*=0.37; **Supplementary Table S3**).

### 3.5 Low mean and minimum tacrolimus trough levels are linked to poor long-term outcomes

A mean tacrolimus trough level below 7 ng/mL was observed in 86 patients (23.1%, **Supplementary Figure S3A**) and was significantly associated with poorer 5-year death-censored graft survival (*P*=0.002, **Supplementary Figure S3B**). In addition, mean tacrolimus levels below 7 ng/mL were significantly associated with poorer overall graft survival and worse patient survival (**Supplementary Figures S4A, B**). No significant associations were observed with DSA-free or rejection-free survival (**Supplementary Figures S4C, D**).

Similarly, minimum tacrolimus trough levels <5 ng/mL were strongly associated with adverse long-term outcomes, including reduced overall graft survival, patient survival, and rejection-free survival (**Supplementary Figures S5**, **S6**).

Consistent with unadjusted analyses, multivariable Cox regression confirmed that minimum tacrolimus trough levels <5 ng/mL were independently associated with significantly increased risks of overall graft failure (HR 3.18, 95% CI 1.77–5.71; *P*<0.001), death-censored graft failure (HR 4.01, 95% CI 2.07–7.77; *P*<0.001), patient death (HR 3.44, 95% CI 1.31–9.01; *P*=0.012), and biopsy-proven rejection (HR 2.45, 95% CI 1.32–4.55; *P*=0.005). No independent association was observed with dnDSA development (HR 0.78, 95% CI 0.44–1.37; *P*=0.38; **Supplementary Table S4**).

## 4 Discussion

In this study, we evaluated various tacrolimus exposure metrics during the early post-transplant period (3–6 months) in a real-world cohort of kidney transplant recipients. Our findings underscore the clinical relevance of multiple tacrolimus exposure metrics in this early period and their distinct associations with long-term kidney transplant outcomes. Specifically, both high intrapatient variability (IPV ≥30%) and pronounced fluctuations in trough levels (maximum/minimum quotient ≥3.0) were independently associated with a significantly increased risk of biopsy-proven rejection (HR 2.40; 95% CI: 1.25–4.60; *P*=0.009 for high IPV and HR 2.32; 95% CI: 1.19–4.53; *P*=0.014 for high quotient). In addition, both low minimum tacrolimus trough levels (<5 ng/mL) and prolonged subtherapeutic exposure (<6 ng/mL; normalized AUC ≥0.2) were independently associated with significantly worse graft and patient survival. More specifically, low minimum trough levels were linked to increased risks of overall graft failure (HR 3.18; 95% CI: 1.77–5.71; *P*<0.001) and patient death (HR 3.44; 95% CI: 1.31–9.01; *P*=0.012), while an elevated AUC below the therapeutic threshold was similarly associated with higher risks of graft loss (HR 3.40; 95% CI: 1.91–6.03; *P*<0.001) and mortality (HR 4.05; 95% CI: 1.60–10.3; *P*=0.003).

Our findings align with and reinforce prior evidence supporting the prognostic value of tacrolimus exposure variability in kidney transplantation. As comprehensively reviewed by Gonzales et al. (22), numerous studies have consistently shown that high tacrolimus IPV is independently associated with adverse long-term outcomes, including graft failure, acute rejection, and the development of DSAs. Borra et al. (29) were the first to demonstrate a clear link between high IPV and poor composite graft outcomes, results that were later confirmed by Shuker et al. (28). Analyses by O’Regan et al. (35), Rozen-Zvi et al. (36), and Rahamimov et al. (37) have validated these associations across various IPV definitions and time frames. The use of time-weighted coefficient of variation (TWCV), in particular, has enabled earlier detection of clinically meaningful variability, supporting the concept that early fluctuations in tacrolimus exposure may already influence long-term outcomes (36). Furthermore, high IPV has been associated with acute rejection [Ro et al. (38), Whalen et al. (26)], dnDSA formation [Rodrigo et al. (32)], and progressive histologic injury [Vanhove et al. (27)], even in the absence of differences in mean tacrolimus concentrations. Collectively, these studies highlight that stability of exposure, not merely achieving target trough levels, is critical for optimizing long-term graft outcomes. Our study adds to this evolving body of evidence by demonstrating that even during the early post-transplant period (3–6 months), instability in tacrolimus exposure, whether reflected by high IPV, marked fluctuations in trough levels, or prolonged subtherapeutic exposure, is already predictive of key clinical endpoints, including rejection, graft failure, and mortality. Importantly, whereas most previous studies have assessed IPV beyond 6 months post-transplant, our findings underscore that clinically meaningful variability is evident already within the first 3–6 months post-transplant. This early window may offer a critical opportunity for timely intervention, enabling clinicians to reinforce adherence strategies, intensify monitoring, and potentially mitigate long-term risk.

Notably, tacrolimus underexposure was associated with multiple adverse outcomes beyond rejection, and the relatively late median time to death in our cohort (approximately five years post-transplant) suggests that early underexposure is unlikely to drive graft failure and mortality through acute mechanisms alone but may contribute to long-term adverse outcomes via cumulative graft injury, progressive graft dysfunction, and downstream complications. Although histopathological data on chronic allograft injury were not available in our cohort, substantial evidence supports a mechanistic link between tacrolimus exposure instability and progressive structural graft damage. In a large protocol-biopsy study, Egeland et al. demonstrated that high tacrolimus clearance, predisposing to transient under-immunosuppression, was independently associated with the development of interstitial fibrosis and tubular atrophy within the first year after kidney transplantation (39). Similarly, underexposure may allow for intermittent alloimmune activation, contributing to progressive fibrotic remodeling of the graft (40). In addition, Vanhove et al. showed that high tacrolimus IPV was also associated with accelerated progression of chronic histologic lesions, including fibrosis and tubular atrophy, even in the absence of early deterioration in graft function (27). Notably, histologic injury may even precede measurable functional decline (27), highlighting the potential for interventions targeting early tacrolimus exposure instability to mitigate cumulative, subclinical graft damage. Within this established framework, the associations observed in our study between early tacrolimus underexposure, exposure variability, and late graft failure are biologically plausible and likely reflect progressive injury rather than isolated acute events.

Despite the consistent associations observed between several tacrolimus exposure metrics and rejection as well as graft and patient survival, no significant association was found between tacrolimus variability or underexposure and the development of dnDSA in our study. Several factors may explain this finding. First, the number of dnDSA events in the cohort was relatively limited, which may have reduced statistical power to detect moderate associations. Second, the follow-up period for dnDSA assessment may have been insufficient to fully capture late antibody formation, which frequently occurs beyond the early post-transplant phase. Third, dnDSA development is influenced by multiple immunological and non-immunological factors beyond tacrolimus exposure alone, including for example HLA mismatch burden and epitope load (41, 42). Finally, variability in the timing and sensitivity of DSA detection in routine clinical practice may have contributed to the underestimation of dnDSA incidence.

In the context of our study findings, understanding the underlying causes of high IPV is critical. Medication nonadherence is often considered as the primary driver of elevated tacrolimus IPV and is arguably the most modifiable risk factor influencing transplant outcomes (22). However, evidence supporting a direct relationship between nonadherence and IPV remains conflicting. In a strictly monitored, highly adherent cohort studied by Leino et al., tacrolimus exposure was generally stable, with a median CV of 15.2% and only rare occurrence of high IPV, suggesting that in the absence of nonadherence, tacrolimus exhibits relatively predictable pharmacokinetics (43). In contrast, Gokoel et al. and Ko et al. found no significant association between the IPV and adherence in stable kidney transplant populations, using electronic monitoring and validated self-report instruments to assess adherence (44, 45). In both studies, IPV was comparable between adherent and nonadherent patients, even though intermittent nonadherence was relatively common (44, 45). These inconsistencies across studies may reflect differences in patient selection, monitoring periods, or adherence definitions, but they also may underscore that tacrolimus IPV represents a multifactorial interplay, in which nonadherence is only one of several contributors. Nevertheless, unlike genetic polymorphisms or pharmacokinetic variability driven by drug interactions or gastrointestinal factors, nonadherence directly reflects patient behavior and therefore presents a critical opportunity for early intervention. In this context, monitoring torque teno virus loads (TTVL) may offer an additional, indirect measure of immunosuppressive burden (46) and, by extension, also patient adherence. As TTVL has been shown to correlate with the overall level of immunosuppression (30, 47, 48), it could serve as a valuable surrogate marker for both tacrolimus exposure and adherence—analogous to HbA1c as a marker of glycemic control in diabetes management.

To improve adherence, one commonly proposed strategy is to simplify dosing regimens, for example by switching to once-daily tacrolimus formulations. In a comprehensive systematic review, Ho et al. demonstrated that once-daily and twice-daily tacrolimus regimens were comparable in terms of biopsy-proven rejection, graft survival, and patient survival up to 12 months post-transplant (49). However, evidence that once-daily dosing improves tacrolimus exposure stability is limited. In a randomized cross-over trial, Bunthof et al. found no significant reduction in IPV after conversion to once-daily tacrolimus, despite potential adherence-related benefits (50). Similar findings were reported in other studies, which consistently showed no meaningful change in IPV following conversion (51, 52). Although modest reductions in IPV have been reported in some earlier studies (53, 54), these improvements were small, inconsistently reproduced, and unlikely to be clinically meaningful (50). Collectively, these findings indicate that simplifying the dosing regimen alone may not be sufficient to reduce IPV, and that improving exposure stability likely requires targeting behavioral or clinical factors beyond the dosing schedule.

Building on our findings that early exposure instability, reflected by high IPV, large fluctuations in trough levels, and prolonged periods below the therapeutic range, is strongly linked to rejection, graft loss, and mortality, it seems essential to address the sources of interindividual variability from the outset. While nonadherence remains a well-recognized and modifiable factor, pharmacogenetic differences also contribute substantially to variability in tacrolimus exposure. Among these, variation in the *CYP3A5* gene plays a central role. Individuals with the CYP3A5 *1/*1 or *1/*3 genotype (referred to as expressers) exhibit significantly lower dose-adjusted tacrolimus trough concentrations compared to non-expressers (CYP3A5 *3/*3). As a result, carriers of the CYP3A5*1 allele typically require 1.5 to 2 times higher doses to achieve comparable blood levels (55). This polymorphism alone is estimated to account for up to 45% of the variability in tacrolimus dose requirements (56) and has therefore been incorporated into dosing guidelines by the Clinical Pharmacogenetics Implementation Consortium (CPIC) (55). Although routine genotyping is not yet standard practice in many transplant centers, evidence from clinical trials, such as the first randomized controlled trial by Thervet et al., demonstrates that genotype-guided dosing can help achieve therapeutic tacrolimus levels more quickly in the early post-transplant period (57). Yet, two other randomized controlled trials did not demonstrate a clear clinical benefit from using *CYP3A5* genotype alone to guide the initial tacrolimus dose (58, 59). More recently, a single-arm study by Francke et al. using an algorithm that included *CYP3A4/CYP3A5* genotypes, body surface area, and age showed that 58% of patients reached target tacrolimus levels by day 3, highlighting the potential advantage of multifactorial models over genotype alone (60).

In parallel, other model-informed precision dosing approaches, such as population pharmacokinetic (PPK) modeling and Bayesian prediction, have shown promise in optimizing initial dose selection, reducing IPV, and minimizing the need for subsequent dose adjustments (61). In a prospective trial, PPK-guided dosing was associated with earlier achievement of target tacrolimus trough levels, fewer dose adjustments, and lower IPV compared with standard weight-based dosing, although no differences in short-term clinical outcomes were observed (62). These findings support the potential of algorithm-based dosing strategies to improve early tacrolimus management. Nevertheless, a substantial proportion of patients remained outside the therapeutic range, underscoring the need for further refinement through advanced modeling approaches, including artificial intelligence and expanded pharmacogenetic integration, to advance individualized immunosuppression (63).

While our findings highlight the adverse consequences of tacrolimus underexposure, particularly prolonged time below therapeutic thresholds, they do not address the potential countervailing risks associated with over-immunosuppression. Potent immunosuppression increases susceptibility to serious infections, which remain the leading non-cardiovascular cause of death in kidney transplant recipients (64, 65). Achieving the right balance of immunosuppression remains a critical clinical challenge, as both under- and over-immunosuppression can lead to serious complications, including rejection, infection, and drug-related toxicity that adversely affect graft and patient survival. Importantly, the tacrolimus exposure metrics evaluated in this study primarily capture risks related to immunosuppression underexposure and are not suited to quantify the risks of over-immunosuppression. Biomarkers reflecting the net state of immunosuppression, such as torque teno virus loads, may complement tacrolimus exposure metrics by providing insight into overall immunocompetence and infection risk (46). As this study did not include a systematic assessment of infectious complications or other outcomes related to over-immunosuppression, we were unable to formally evaluate infection risk across exposure groups. Clinicians should therefore interpret our findings within the broader framework of individualized immunosuppressive management, integrating tacrolimus exposure with immunological risk, infection history, and overall clinical vulnerability rather than focusing solely on maintaining drug levels above a fixed threshold.

In summary, our study demonstrates that early instability in tacrolimus exposure, reflected by high intrapatient variability, large fluctuations in trough levels, and prolonged subtherapeutic concentrations, is strongly associated with increased risks of rejection, graft loss, and mortality. Among the strengths of this study are its large real-world cohort, the use of multiple validated tacrolimus exposure metrics, and robust multivariable adjustment for potential confounders. However, our study has several important limitations: (i) the retrospective, single-center design may limit generalizability, and the possibility of residual confounding cannot be excluded. Nonetheless, the real-world nature of the cohort and the consistency of associations across multiple tacrolimus exposure metrics support the robustness of the observed findings. (ii) Although this represents one of the larger real-world analyses of early tacrolimus exposure variability, the sample size may limit power for less frequent outcomes, particularly *de novo* donor-specific antibody formation. (iii) Restricting inclusion to recipients with a functioning graft at day 180 may introduce survival bias by excluding early graft losses, some of which may have been related to severe tacrolimus underexposure. This design choice was intentional, as early graft loss is often driven by perioperative complications or rejection episodes requiring rapid immunosuppressive dose adjustments (66), resulting in unstable tacrolimus exposure that precludes meaningful assessment of maintenance exposure metrics. Moreover, stable graft function beyond day 180 was necessary to enable subsequent longitudinal outcome analyses. (iv) Finally, tacrolimus IPV was assessed using the standard coefficient of variation rather than time-weighted measures. Therefore, future studies are warranted that consider multicenter cohorts, longer follow-up, and advanced pharmacokinetic modeling, including time-weighted variability metrics, to further validate and refine risk stratification strategies.

Beyond our own data, we also briefly discuss additional contributors to tacrolimus variability and emerging strategies for individualized management in clinical practice. Nonadherence remains an important and modifiable contributor, with once-daily formulations representing one potential strategy to support adherence. Furthermore, growing evidence supports the integration of pharmacogenetic information, particularly based on the *CYP3A5* genotype, into tacrolimus dosing, an approach already reflected in the current CPIC guidelines and likely to become increasingly feasible as genetic testing becomes more accessible. In addition, monitoring torque teno virus loads as dynamic indicators of overall immunosuppressive burden may also provide insights into patient adherence. Ultimately, optimizing tacrolimus exposure to preserve long-term graft function will likely require a personalized, multifaceted strategy combining pharmacogenetic-guided dosing, adherence support, immunological risk stratification, and biomarker-informed monitoring.

## Data Availability

The raw data supporting the conclusions of this article will be made available by the authors, without undue reservation.
