# Supplementary material for: The importance of staying within range: associations between tacrolimus intrapatient variability and kidney transplant outcomes
Source: Front Immunol. 2026 Jan 29;17:1739104. doi: 10.3389/fimmu.2026.1739104 (PMC12894007; doi:10.3389/fimmu.2026.1739104)
Supplement: Supplementary file 1 [file DataSheet1.docx]

Supplementary Material

# Table of Contents

**Supplementary Table S1** Risk Estimates for Key Clinical Outcomes in Patients with Tacrolimus Intrapatient Variability ≥30%

**Supplementary Table S2** Risk Estimates for Key Clinical Outcomes in Patients with Tacrolimus Maximum/Minimum Quotient ≥3

**Supplementary Table S3** Risk Estimates for Key Clinical Outcomes in Patients with a normalized AUC ≥ 0.2 for Trough Levels <6ng/mL

**Supplementary Table S4** Risk Estimates for Key Clinical Outcomes in Patients with Low Tacrolimus Minimum Values (<5ng/mL)

**Supplementary Figure S1** Patient Selection Flowchart

**Supplementary Figure S2** Number of Tacrolimus Trough Level Values per Patient and Five-Year Death-Censored Graft Survival

**Supplementary Figure S3** Mean Tacrolimus Trough Level Values per Patient and Five-Year Death-Censored Graft Survival

**Supplementary Figure S4** Mean Tacrolimus Trough Level Values per Patient and Overall Graft Survival, Patient Survival, DSA-Free Survival and Rejection-Free Survival

**Supplementary Figure S5** Minimum Tacrolimus Trough Levels and Five-Year Death-Censored Graft Survival

**Supplementary Figure S6** Minimum Tacrolimus Trough Levels and Overall Graft Survival, Patient Survival, DSA-Free Survival and Rejection-Free Survival**Supplementary Table S1** Risk Estimates for Key Clinical Outcomes in Patients with Tacrolimus Intrapatient Variability ≥30%

| **Coefficient of Variation ≥ 30%** | **N** | **Hazard Ratio** | **95% CI** | **Log-rank *P*-value** |
| --- | --- | --- | --- | --- |
| Overall graft failure | 311 | 1.07 | 0.56 – 2.04 | 0.84 |
| Death-censored graft failure | 311 | 1.33 | 0.67 – 2.65 | 0.42 |
| Patient death | 311 | 0.57 | 0.18 – 1.80 | 0.34 |
| **Rejection** | **274** | **2.40** | **1.25 – 4.60** | **0.009^(**)^** |
| DSA | 240 | 1.04 | 0.51 – 2.12 | 0.92 |

Multivariable Cox regression analysis assessing the association between high Tacrolimus intrapatient variability (coefficient of variation ≥30%) and five key transplant outcomes: overall graft failure, death-censored graft failure, patient death, biopsy-proven rejection, and development of donor-specific antibodies (DSA). Results are expressed as hazard ratios (HR) with 95% confidence intervals (CI) and corresponding *P*-values. Statistically significant associations (*P*<0.05) are indicated. CI, confidence interval; DSA, donor-specific antibodies; N, number; ***P*<0.01

**Supplementary Table S2** Risk Estimates for Key Clinical Outcomes in Patients with Tacrolimus Maximum/Minimum Quotient ≥3

| **Quotient ≥ 3** | **N** | **Hazard Ratio** | **95% CI** | **Log-rank *P*-value** |
| --- | --- | --- | --- | --- |
| Overall graft failure | 311 | 1.90 | 1.00 – 3.61 | 0.050 |
| Death censored graft failure | 311 | 1.90 | 0.95 – 3.81 | 0.069 |
| Patient death | 311 | 1.19 | 0.32 – 4.35 | 0.79 |
| **Rejection** | **274** | **2.32** | **1.19 – 4.53** | **0.014^(*)^** |
| DSA | 240 | 0.92 | 0.42 – 2.00 | 0.83 |

Multivariable Cox regression analysis assessing the association between elevated Tacrolimus maximum/minimum quotient (Quotient ≥3.0) and five key transplant outcomes: overall graft failure, death-censored graft failure, patient death, biopsy-proven rejection, and development of donor-specific antibodies (DSA). Results are expressed as hazard ratios (HR) with 95% confidence intervals (CI) and corresponding *P*-values. Statistically significant associations (*P*<0.05) are indicated. CI, confidence interval; DSA, donor-specific antibodies; N, number; **P*<0.05

**Supplementary Table S3** Risk Estimates for Key Clinical Outcomes in Patients with a normalized AUC ≥ 0.2 for Trough Levels <6ng/mL

| **AUC ≥ 0.2** | **N** | **Hazard Ratio** | **95% CI** | **Log-rank *P*-value** |
| --- | --- | --- | --- | --- |
| **Overall graft failure** | **372** | **3.40** | **1.91 – 6.03** | **<0.001^(***)^** |
| **Death censored graft failure** | **372** | **4.03** | **2.13 – 7.60** | **<0.001^(***)^** |
| **Patient death** | **372** | **4.05** | **1.60 – 10.3** | **0.003^(**)^** |
| Rejection | 333 | 1.47 | 0.75 – 2.87 | 0.26 |
| DSA | 283 | 0.74 | 0.38 – 1.44 | 0.37 |

Multivariable Cox regression analysis assessing the association between high Tacrolimus area under the curve under 6 (AUC ≥0.2) and five key transplant outcomes: overall graft failure, death-censored graft failure, patient death, biopsy-proven rejection, and development of donor-specific antibodies (DSA). Results are expressed as hazard ratios (HR) with 95% confidence intervals (CI) and corresponding *P*-values. Statistically significant associations (*P*<0.05) are indicated. CI, confidence interval; DSA, donor-specific antibodies; N, number; ****P*<0.001; **P*<0.05

**Supplementary Table S4** Risk Estimates for Key Clinical Outcomes in Patients with Low Tacrolimus Minimum Values (<5ng/mL)

| **Minimum < 5ng/mL** | **N** | **Hazard Ratio** | **95% CI** | **Log-rank *P*-value** |
| --- | --- | --- | --- | --- |
| **Overall graft failure** | **372** | **3.18** | **1.77 – 5.71** | **<0.001^(***)^** |
| **Death censored graft failure** | **372** | **4.01** | **2.07 – 7.77** | **<0.001^(***)^** |
| **Patient death** | **372** | **3.44** | **1.31 – 9.01** | **0.012^(*)^** |
| **Rejection** | **333** | **2.45** | **1.32 – 4.55** | **0.005^(**)^** |
| DSA | 283 | 0.78 | 0.44 – 1.37 | 0.38 |

Multivariable Cox regression analysis assessing the association between low Tacrolimus minimum trough levels (Minimum <5ng/mL) and five key transplant outcomes: overall graft failure, death-censored graft failure, patient death, biopsy-proven rejection, and development of donor-specific antibodies (DSA). Results are expressed as hazard ratios (HR) with 95% confidence intervals (CI) and corresponding *P*-values. Statistically significant associations (*P*<0.05) are indicated. CI, confidence interval; DSA, donor-specific antibodies; N, number; ****P*<0.001; ***P*<0.01; **P*<0.05

**Supplementary Figure S1** Patient Selection Flowchart


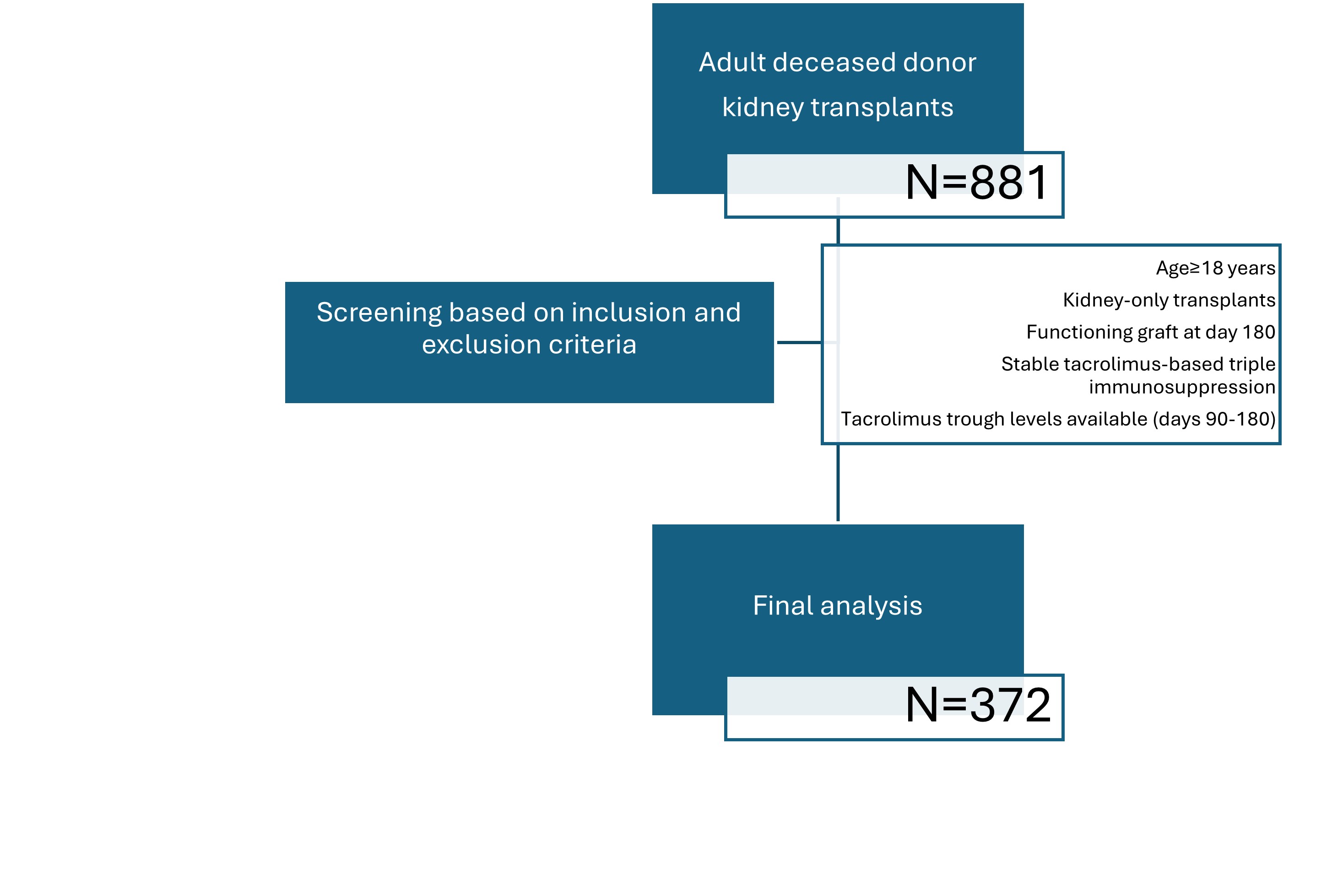


**Legend to Supplementary Figure S1** Flowchart illustrating patient screening, inclusion and exclusion leading to the final study cohort of 372 kidney transplant recipients

**Supplementary Figure S2** Number of Tacrolimus Trough Level Values per Patient and Five-Year Death-Censored Graft Survival


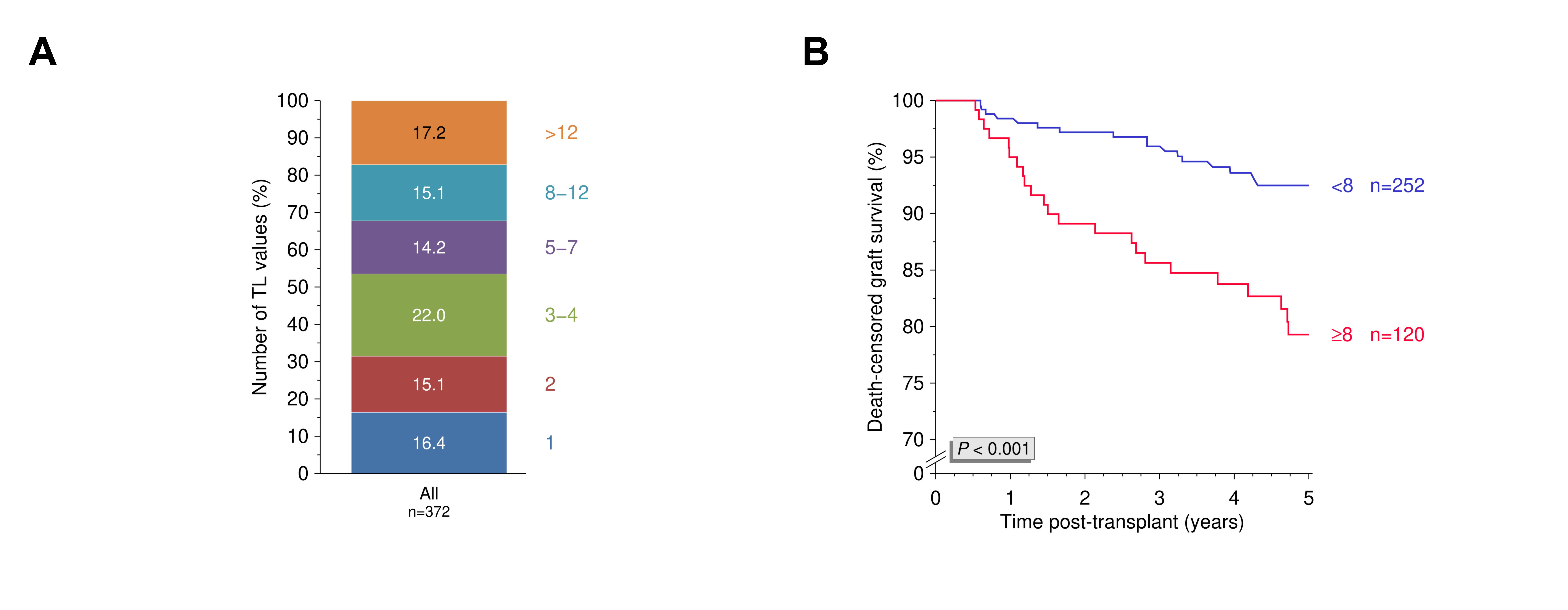


**Legend to Supplementary Figure S2** (A) Distribution of available tacrolimus trough levels between days 90 and 180 post-transplant in the study cohort. (B) Five-year death-censored graft survival in patients with <8 tacrolimus trough levels (N=252) and ≥8 tacrolimus trough levels (N=120).

N, number; TL, trough level

**Supplementary Figure S3** Mean Tacrolimus Trough Level Values per Patient and Five-Year Death-Censored Graft Survival


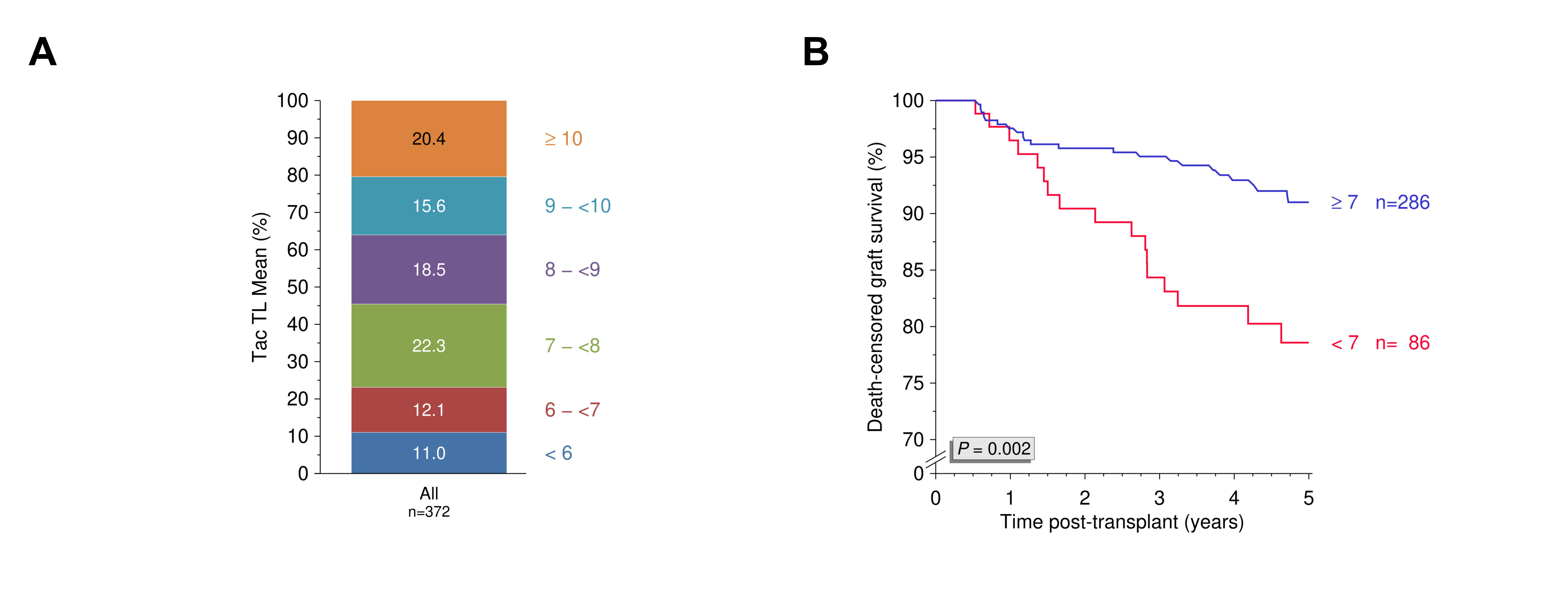


**Legend to Supplementary Figure S3** (A) Distribution of mean tacrolimus trough levels per patient between days 90 and 180 post-transplant in the study cohort. (B) Five-year death-censored graft survival in patients with mean tacrolimus trough levels ≥7ng/mL (N=286) and <7ng/mL (N=86).

N, number; Tac, tacrolimus; TL, trough level

**Supplementary Figure S4** Mean Tacrolimus Trough Level Values per Patient and Overall Graft Survival, Patient Survival, DSA-Free Survival and Rejection-Free Survival



**Legend to Supplementary Figure S4** Kaplan–Meier analyses of (A) overall graft survival, (B) patient survival, (C) DSA-free survival, and (D) rejection-free survival in patients stratified by mean tacrolimus trough level ≥7 and <7 ng/mL.

DSA, donor-specific antibodies; N, number

**Supplementary Figure S5** Minimum Tacrolimus Trough Levels and Five-Year Death-Censored Graft Survival


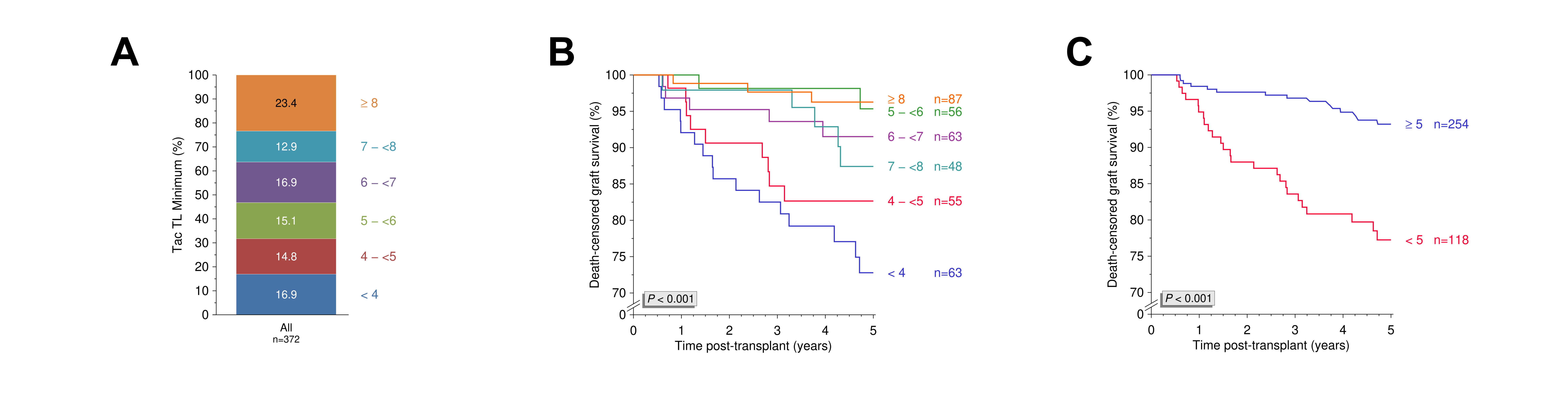


**Legend to Supplementary Figure S5** (A) Distribution of minimum tacrolimus trough levels recorded between days 90 and 180 post-transplant in the study cohort. (B) Five-year death-censored graft survival across different minimum tacrolimus trough levels with identification of a clinically relevant minimum tacrolimus trough level <5 ng/mL. (C) Five-year death-censored graft survival in patients with a minimum tacrolimus trough level ≥5 ng/mL (N=254) and <5ng/mL (N=118).

N, number; Tac, tacrolimus; TL, trough level

**Supplementary Figure S6** Minimum Tacrolimus Trough Levels and Overall Graft Survival, Patient Survival, DSA-Free Survival and Rejection-Free Survival





**Legend to Supplementary Figure S6** Kaplan–Meier analyses of (A) overall graft survival, (B) patient survival, (C) DSA-free survival, and (D) rejection-free survival in patients with minimum tacrolimus trough levels <5 and ≥5ng/mL.

DSA, donor-specific antibodies; N, number
